# Dual Diagnosis: Unraveling Ankylosing Spondylitis in a Cancer Patient

**DOI:** 10.7759/cureus.72248

**Published:** 2024-10-24

**Authors:** Liana Galimova, Rustem Safin

**Affiliations:** 1 General Practice, KazanOncoNet, Kazan, RUS; 2 Internal Medicine, Kazan Federal University Institute of Fundamental Medicine and Biology, Kazan, RUS; 3 Chemotherapy, Republican Clinical Oncology Dispensary named after Prof. M.Z. Sigal, Kazan, RUS

**Keywords:** ankylosing spondylitis, autoimmune disease, breast cancer, chemotherapy, psoriasis

## Abstract

There have been several recent studies that indicate that patients with rheumatological diseases are at a higher risk of developing malignant neoplasms than the general population. This clinical case presents a patient with ankylosing spondylitis, a rare autoimmune disease in women, in addition to breast cancer. The close relationship between rheumatology and malignancy highlights the challenges in terms of diagnosis, treatment, and prevention, making this case highly relevant. Furthermore, this case also highlights the positive effect of chemotherapy drugs used in the treatment of breast cancer on the autoimmune disease. Therefore, further research in this area is necessary to gain a better understanding of the complex interplay between rheumatology and malignancy.

## Introduction

Ankylosing spondylitis has historically been regarded as a predominantly male-affecting disease. However, recent literature indicates a higher incidence among women in contemporary studies. The male/female ratio varies, with data suggesting a distribution of 2-3:1 [1]. In this clinical case report, we discuss the factors that complicated the diagnosis of ankylosing spondylitis. In recent years, there have been discussions regarding the common etiological factors and genetic predisposition in the development of rheumatological and oncological diseases [2]. Although the etiology and pathogenesis of these diseases are complex and not fully understood, common causes include infectious (viral), chemical, immunological, genetic, and other factors [3-5]. In this case, Epstein-Barr virus (EBV), human herpes virus type 6 (HHV-6), and cytomegalovirus (CMV) were detected. For instance, in rheumatology, EBV is considered a possible trigger of systemic rheumatic diseases, while in oncology, it predisposes patients to the development of Burkitt's lymphoma and lymphogranulomatosis [1]. The presented clinical case is also interesting as it provides an opportunity to study the positive effects of chemotherapy for cancer on autoimmune diseases, which is a subject of current worldwide research [6].

## Case presentation

We present the case of a 35-year-old Caucasian female patient, a self-employed entrepreneur, who was diagnosed with ankylosing spondylitis one year ago, but we believe that the disease began in adolescence, approximately 20 years ago, when she experienced periodic back pain that could not be associated with any cause. Despite being scheduled for a rheumatology consultation, the patient did not attend the appointment.

The patient had been actively involved in ballroom dancing since childhood, which may have slowed the progression of the disease. She experienced periodic back pain for approximately two decades, usually in the morning after waking up, which she successfully managed with over-the-counter nonsteroidal anti-inflammatory drugs (NSAIDs) and did not seek medical attention.

**Figure 1:**
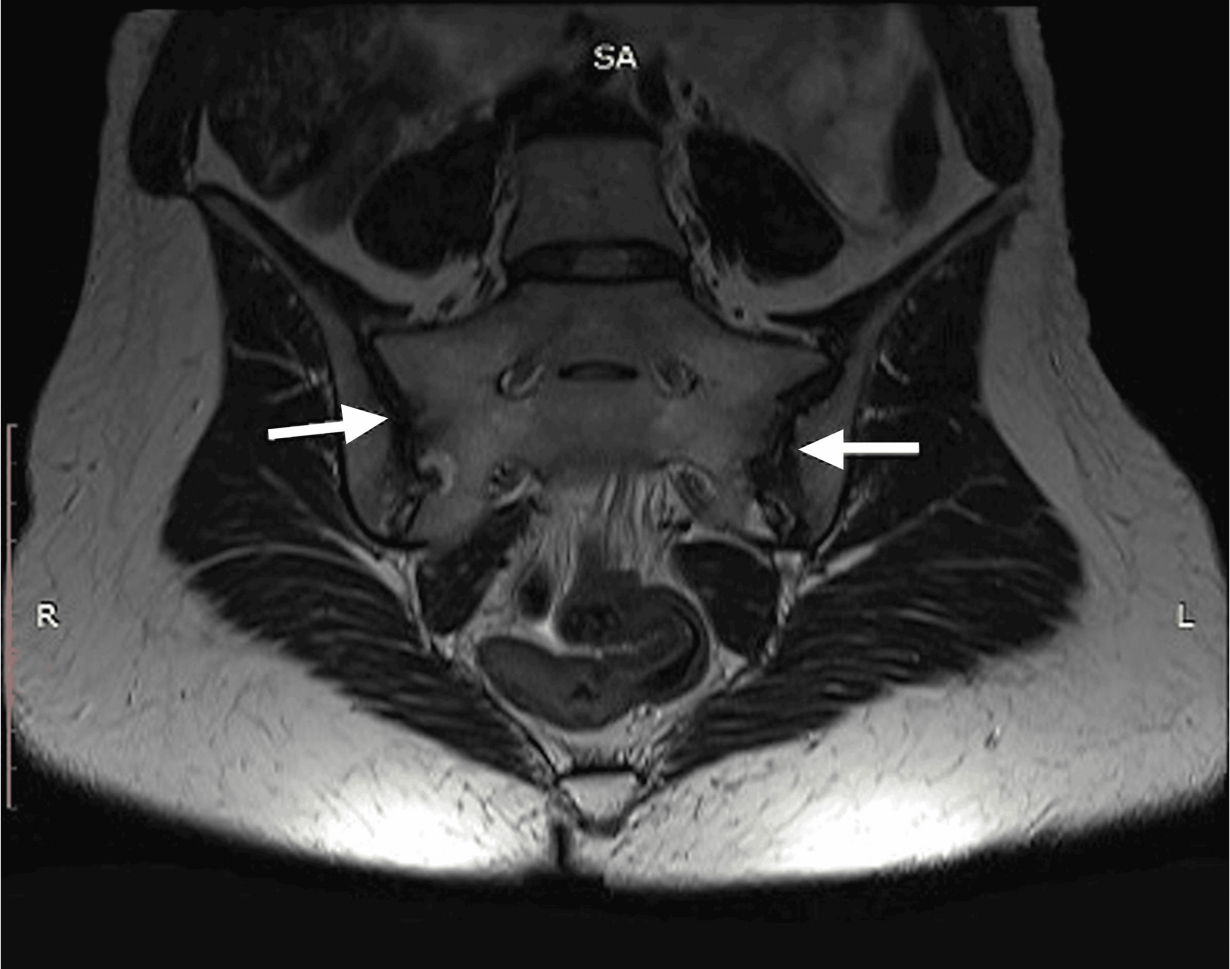
MRI of sacroiliac joints MRI conclusion: The joint space is seen throughout. Moderate bilateral joint space widening. The articular surfaces on both sides are clear and uneven with the presence of subchondral sclerosis. Some very subtle defects in the lower third of the joints are seen on both sides. Trabecular edema is present on the subchondral sections on both sides, on the right lower third of the joint, and on the left throughout. MRI: magnetic resonance imaging

About 13 years ago, polymerase chain reaction (PCR) tests revealed the presence of Epstein-Barr virus, cytomegalovirus, and herpes simplex virus 6, which could trigger the development of autoimmune and/or oncological processes in the body [3,7,8]. Ankylosing spondylitis was suspected because of periodic back pain and frequent episodes of iridocyclitis, and after the third episode of iridocyclitis, she was referred to a rheumatologist who ordered several tests. The test for human leukocyte antigen B27 (HLA-B27) was positive, and magnetic resonance imaging (MRI) suggested acute bilateral sacroiliitis (Figure 1). Based on her symptoms and these findings, ankylosing spondylitis was diagnosed.

**Figure 2:**
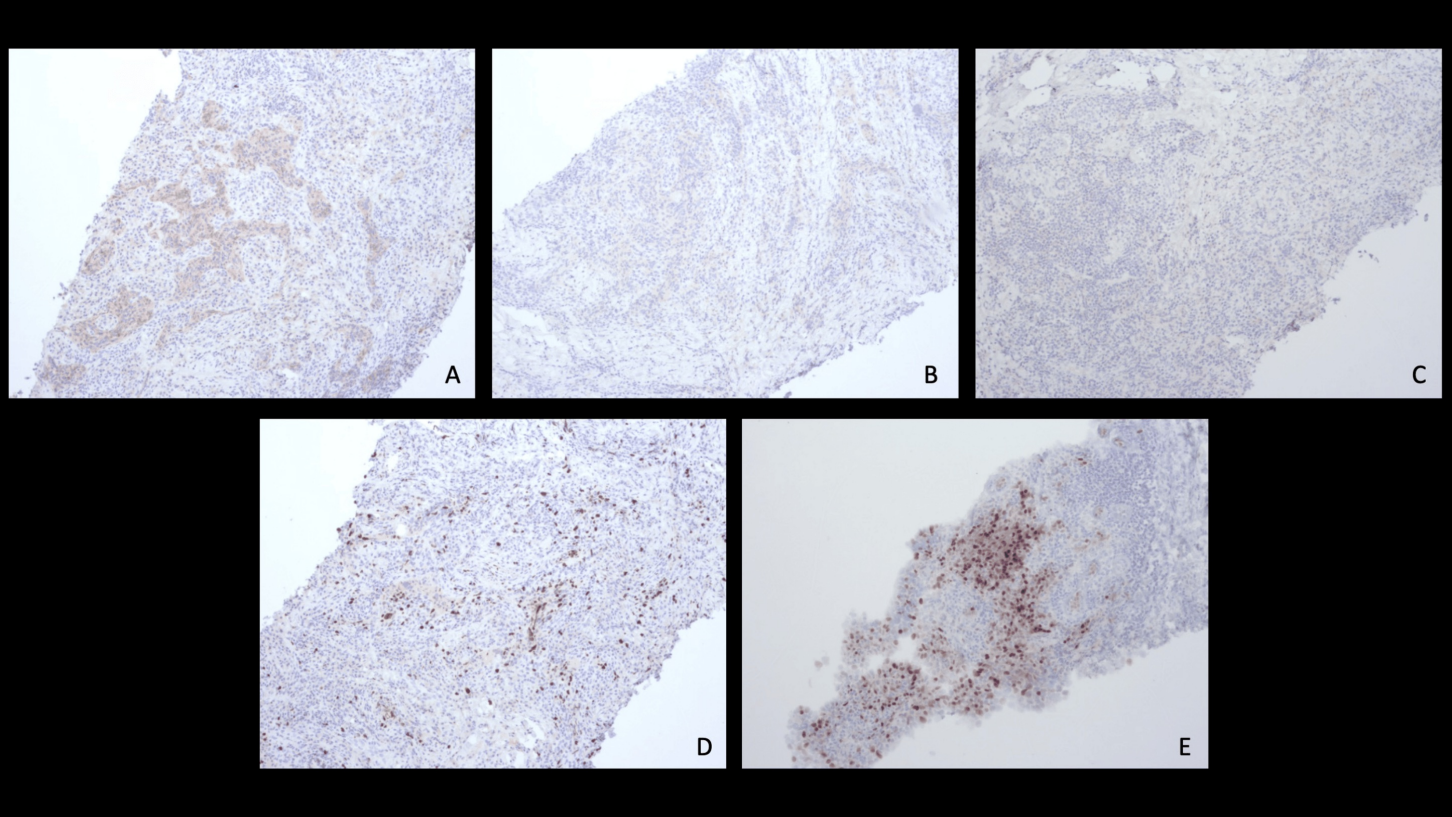
(A) HER2 immunohistochemistry at 10× magnification, (B) ER immunohistochemistry at 10× magnification, (C) PR immunohistochemistry at 10× magnification, (D) Ki67 immunohistochemistry at 10× magnification, and (E) AR immunohistochemistry at 10× magnification All immunohistochemical stainings were done using hematoxylin and eosin. HER2: human epidermal growth factor receptor 2, ER: estrogen receptor, PR: progesterone receptor, AR: androgen receptor

At the age of 35, the patient reported experiencing a painful lump in the right breast, measuring 1.5 × 1.0 cm. The patient waited for about three months to see if it resolves on its own, and when it did not, the patient consulted a mammologist. Given the suspicion of malignancy, the patient was referred to the oncology center for further examination. A histological examination of the tumor revealed invasive cancer of the right breast with high malignancy, with a score of 8 (3+3+3) according to the Nottingham grading system (G3). The tumor stroma demonstrated dense mononuclear infiltration. Immunohistochemistry (IHC) results were positive for indoleamine 2,3-dioxygenase (IDO) 5260 and negative for human epidermal growth factor receptor 2 (HER2) (Figure 2), estrogen receptors (ER) (Figure 2A), and progesterone receptors (PR) (Figure 2C). The Ki67 index was found to be 36% (Figure 2D). An uneven positive reaction to ACK 5 in cancer with basal-like differentiation was observed. Androgen receptor (AR) was 6 (40%), with focal signs of apocrine differentiation (Figure 2E). The TNM diagnosis was T1cNxMo, stage 2. A negative result was obtained in the genomic sequencing (NGS) analysis of mutations in the *BRCA1* and *BRCA2* genes.

The patient was prescribed a chemotherapy regimen in accordance with current clinical recommendations, which comprised the following regimen: doxorubicin/cyclophosphamide (AC) × 4 → (paclitaxel (P) + carboplatin (carbo)) × 12 [9]. The dosage of doxorubicin was 60 mg/m² on day 1, and cyclophosphamide was administered at a dose of 600 mg/m² on the same day; the treatment cycle was 14 days. The paclitaxel dose was 80 mg/m² on days 1, 8, and 15, and carboplatin was administered at a dose of AUC 6 on day 1, with the treatment cycle lasting 21 days.

Just before the commencement of chemotherapy, the patient reported experiencing skin redness, peeling, and itching, which might have been an extra-articular manifestation of ankylosing spondylitis (psoriasis) [10]. During the patient interview, it was revealed that the "paternal grandmother had psoriasis, as well as some problems with posture." Although ankylosing spondylitis is difficult to diagnose in women several decades ago, the presence of ankylosing spondylitis in the patient's relative cannot be ascertained accurately due to the lack of data.

Upon the completion of chemotherapy, the ankylosing spondylitis activity was evaluated using some scores and indices, and the results are presented in Table 1.

**Table 1:** Comparison of various scores and indices evaluating diseases activity of ankylosing spondylitis *Evaluation of activity ^Assessment of the safety of functions (AS) #Assessment of peripheral joint and enthesis BASDAI: Bath Ankylosing Spondylitis Disease Activity Index, ASDAS: Ankylosing Spondylitis Disease Activity Score, NRS: numerical rating scale, BASFI: Bath Ankylosing Spondylitis Functional Index, BASMI: Bath Ankylosing Spondylitis Metrology Index, MASES: Maastricht Ankylosing Spondylitis Enthesitis Score

Name of score/index	Reference range	Before chemotherapy	After chemotherapy
BASDAI*	BASDAI score ≥ 4.0 corresponds to high ankylosing spondylitis activity	4.1 (high activity level)	2.7 (low activity level)
ASDAS*	ASDAS < 1.3 corresponds to low activity, ASDAS ≤ 1.3 to <2.1 corresponds to moderate activity, ASDAS ≤ 2.1 to <3.5 corresponds to high activity, ASDAS ≥ 3.5 corresponds to very high activity	2.16 (high activity level)	1.4 (moderate activity level)
BASFI^	BASFI score ≥ 4.0 pronounced functional disorders	3.8	2.3
BASMI^	BASMI ranges from 0 to 10, where 0 is no mobility limitation and 10 is very severe limitation	5 out of 10	4 out of 10
MASES^#^	MASES (maximum is 13)	3 (spinous process of the 5th lumbar vertebra, posterior superior iliac spine)	2 (posterior superior iliac spine)
Number of swollen joints	Assessment of 44 joints	14	10

## Discussion

The misconception that women are less likely to suffer from ankylosing spondylitis than men, combined with the independent and uncontrolled use of NSAIDs by patients and the lack of coordination among various specialists, can all contribute to the late diagnosis of ankylosing spondylitis. However, the literature suggests that systematic exercise can improve the course of ankylosing spondylitis and significantly slow disease progression [11]. It is also essential to carefully examine patients for extra-skeletal manifestations of ankylosing spondylitis, such as psoriasis, inflammatory bowel diseases, and nephropathy [11]. Since chemotherapy cycles influenced the disease course in our case, the benefits/risks analysis is presented in Table 2.

**Table 2:** Comparison of chemotherapy effects in our patient *Probable psoriasis (according to the patient's complaints, there is a history of psoriasis, but during chemotherapy, our patient had no clinical manifestations) NSAIDs: nonsteroidal anti-inflammatory drugs

Duration	Effects of chemotherapy on the patient	Side effects of chemotherapy on the patient
From the first month	Absence of pain at the breast tumor site	Leukopenia, neutropenia, eosinophilia, and basophilia
	Severity of pain in the back and sacroiliac joints decreased, and the patient was free of any NSAIDs	Limitations in daily activities due to increased fatigue, drowsiness, and memory impairment
		Hair loss
	Dermatological manifestations improved*	Gradual weight gain (8 kg gained during the entire course)
From the second month	Complete regression of the lump in the breast (based on physical examination and the results of ultrasound)	Liver dysfunction
		Iron deficiency anemia
		Increased susceptibility to infection (frequent exacerbation of tonsillopharyngitis and urinary tract infection (once))
		Frequent recurrence of shingles (four times during the entire course)

**Figure 3:**
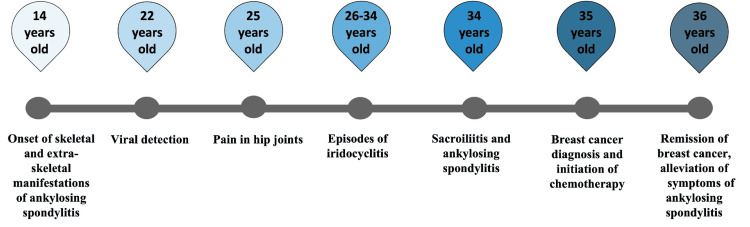
Timeline of the patient's manifestations and interventions

In the presented case, the positive effect of chemotherapy on ankylosing spondylitis was confirmed. In Figure 3, crucial events are displayed using a horizontal timeline in chronological order.

The impact of chemotherapy on autoimmune disorders, such as ankylosing spondylitis, can be attributed to its ability to suppress the production of autoantibodies by plasma cells and memory B-cells. This reduction in autoantibodies results in a decrease in the severity of symptoms associated with the disease. While chemotherapy does have side effects, the complications that may arise can generally be managed and treated. Additionally, some side effects may resolve on their own following the completion of chemotherapy.

## Conclusions

Autoimmune diseases can trigger the development of more aggressive neoplasms. Given the benefit/risk ratio of chemotherapy, it can be stated that in this instance, the treatment achieved two objectives simultaneously: the complete remission of the cancer and the improvement of skeletal and extra-articular symptoms associated with ankylosing spondylitis. Gathering a comprehensive history including family and medical history is of critical importance for early screening and diagnosis. We have reason to believe that the patient's maternal grandmother was suffering from ankylosing spondylitis, a condition that genetically predisposed our patient to the same disease. Additionally, the patient's family medical history is also noteworthy as the paternal grandmother was diagnosed with uterine cancer. In light of these findings, it is recommended that healthcare professionals working with patients diagnosed with autoimmune diseases possess cancer awareness and that consultation with an oncologist should be considered. Furthermore, patients from this category should be advised of their genetic predisposition, particularly with regard to their offspring, as evidenced by the patient's 17-year-old son who exhibits symptoms of stiffness in his back.
